# Comparison of Renal Function Estimation Formulae for Dosing Direct Oral Anticoagulants in Patients with Atrial Fibrillation

**DOI:** 10.3390/jcm8122034

**Published:** 2019-11-21

**Authors:** Kwang-No Lee, Jong-Il Choi, Yun Gi Kim, Ki Yung Boo, Do Young Kim, Yun Young Choi, Ha Young Choi, Dong-Hyeok Kim, Dae In Lee, Seung-Young Roh, Jaemin Shim, Jin Seok Kim, Young-Hoon Kim

**Affiliations:** 1 Division of Cardiology, Department of Internal Medicine, Korea University Medical Center, Seoul 02841, Korea; 2 Division of Cardiology, Department of Internal Medicine, Sejong General Hospital, Bucheon 14754, Korea; 3 Division of Cardiology, Department of Internal Medicine, Chungbuk National University Hospital, Cheongju 28644, Korea

**Keywords:** anticoagulant, warfarin, atrial fibrillation, renal elimination, product labeling

## Abstract

The Cockcroft-Gault (CG) formula is recommended to guide clinicians in the choice of the appropriate dosage for direct oral anticoagulants (DOACs). However, the performance of the CG formula varies depending on the patient’s age, weight, and degree of renal function. We aimed to compare the validity of the CG formula with that of Chronic Kidney Disease Epidemiology Collaboration (CKD-EPI) and Modification of Diet in Renal Disease (MDRD) formulae for dosing DOACs. A total of 6268 consecutive patients on anticoagulants for atrial fibrillation (AF) were retrospectively investigated. Among underweight and elderly patients, the CG formula underestimated renal function compared with the non-CG formulae. However, the concordant rate of drug indications between the CG and the non-CG formulae was approximately 94%. On-label uses under the three formulae were associated with a lower risk of major bleeding (but not thromboembolism) compared to warfarin. Although we found differences in estimating renal function and the proportions of drug indications between the CG and non-CG formulae, the risks of thromboembolism and major bleeding were similar to those with warfarin regardless of which formula was used.

## 1. Introduction

Direct oral anticoagulants (DOACs) have been approved for the prevention of stroke or systemic embolism in patients with non-valvular atrial fibrillation (AF). Phase III trials have demonstrated that DOACs are as effective as dose-adjusted warfarin and have a more favorable safety profile [1,2,3,4]. Thus, the use of DOACs as alternatives to warfarin has increased. Furthermore, the dosing schedules for DOACs are simple and convenient [5].

Plasma concentrations of DOACs in the steady state are determined by renal clearance [6]. In recent guidelines for using DOACs to treat AF, patients with chronic kidney disease require assessment of their renal function to choose the appropriate dosage, measured as the estimated creatinine clearance (CrCl), using the Cockcroft-Gault (CG) formula [7]. However, there are concerns about the accuracy of CrCl estimated using the CG formula, which depends on the patient’s age, body weight, and degree of renal function, even though it is generally used in practice and its clinical utility is supported by evidence from phase III DOAC trials [2,3,4]. The CG formula can underestimate renal function, especially in underweight patients, and that underestimation might be more conspicuous in Asians than in non-Asians [8]. The Modification of Diet in Renal Disease (MDRD) formula is one of the most commonly used formulae for calculating the estimated glomerular filtration rate (eGFR), but it underestimates renal function at high GFRs [9]. Recently, the National Kidney Foundation recommended the Chronic Kidney Disease Epidemiology Collaboration (CKD-EPI) formula for estimating GFR because it offers improved estimation accuracy compared with earlier formulae [10,11].

Accurately assessing renal function is important for appropriately dosing DOACs. Patients on warfarin who have chronic kidney disease are known to have a higher risk of stroke, systemic embolism, and bleeding than those without renal disease [12]. Both underdosing and overdosing of DOACs are associated with an increased risk of adverse events [13]. Therefore, our objectives in this study were to (1) compare the renal functions estimated by different formulae, and (2) determine whether the choice of formula in dosing DOACs could affect clinical outcomes.

## 2. Methods

### 2.1. Study Design

We conducted this retrospective observational study at a tertiary referral center. Drug indications were categorized as on-label reduced, off-label reduced, on-label standard, and off-label standard doses according to the dose and adherence to recommendations in the label approved by the Korean Ministry of Food and Drug Safety (Table A1). Renal function was estimated using the CG, MDRD, and CKD-EPI formulae as follows:

CG, mL/min = (140 − Age) × Weight/(72 × Serum creatinine (SCr)) × (0.85 if female)

MDRD, mL/min/1.73 m^2^ = 175 × SCr^−1.154^ × Age^−0.203^ × (0.742 if female) × (1.210 if African-American)

CKD-EPI, mL/min/1.73 m^2^ = 141 × min (SCr/(0.7 if female; 0.9 if male), 1)^(−0.329 if female; −0.411 if male)^ × max (SCr/(0.7 if female; 0.9 if male), 1)^−1.209^ × 0.993 Age × (1.018 if female) × (1.159 if black)

The outcomes of interest for evaluating the effectiveness of each formula were thromboembolic events (ischemic stroke, systemic thromboembolism, myocardial infarction, and intracardiac thrombus) during anticoagulation therapy. All ischemic strokes were confirmed by diagnostic imaging that excluded the possibility of a transient ischemic attack. Myocardial infarction was diagnosed with typical symptoms, electrocardiographic change, cardiac biomarkers, and coronary intervention. Systemic thromboembolism and intracardiac thrombus were diagnosed with computed tomography. Safety outcomes were adjudicated as overt bleeding events, consistent with the International Society on Thrombosis and Hemostasis definition of major bleeding in non-surgical patients [14].

### 2.2. Data Collection

**Figure 1:**
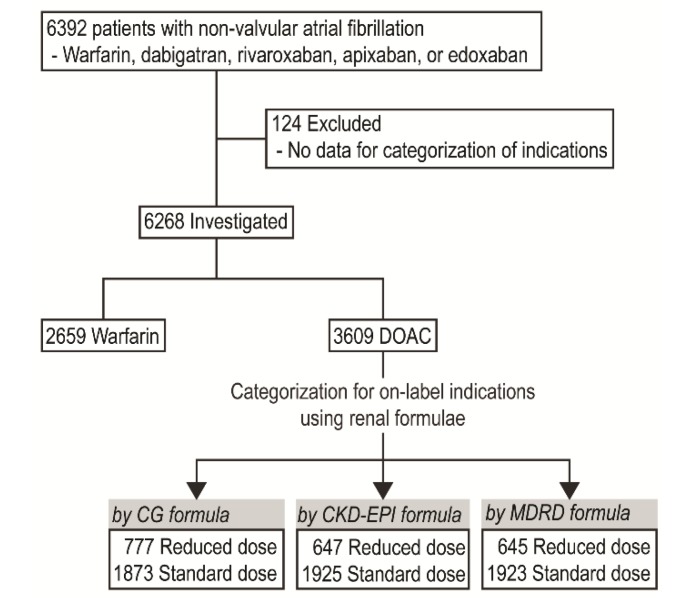
Study flowchart. CG, Cockcroft-Gault formula; CKD-EPI, Chronic Kidney Disease Epidemiology Collaboration formula; DOAC, direct oral anticoagulant; MDRD, Modification of Diet in Renal Disease Study formula.

Data were collected from electronic medical records from January 2013 to June 2018. The Ethics Committee of our hospital’s Institutional Review Board approved this study and waived the need for patient consent. The protocol of this study is consistent with the ethical guidelines of the 2008 Helsinki Declaration. Patients who were prescribed dabigatran, rivaroxaban, apixaban, or edoxaban for AF were screened. We excluded patients who had moderate or severe mitral stenosis, any mechanical heart valve, or no data available to categorize their drug indication according to the formulae we were testing. In the end, a total of 6268 patients (2659 in the warfarin group and 3609 in the DOAC group) were included (Figure 1). SCr was determined using a kinetic modification of the Jaffe procedure.

The duration of anticoagulant therapy was calculated based on the prescription date and total number of days of treatment, excluding those whose treatment was discontinued for more than 30 days. Time-varying drug adherence was estimated as the proportion of days covered (PDC), which we defined as the ratio of the number of days on which a patient was actually on the treatment versus the number of expected days on which the patient should have been given the drug during anticoagulation therapy [15]. Patients with PDC ≥ 80% were considered adherent. 

### 2.3. Statistical Analysis

Continuous variables are described using the mean with standard deviation. Categorical variables are expressed as percentages. Agreement between renal function estimations was assessed using a Bland–Altman plot and intraclass correlation coefficient (ICC) from a one-way random effect model [16]. ICC values of less than 0.4, 0.40–0.59, 0.60–0.74, and 0.75–1.0 indicate poor, moderate, good, and excellent agreement, respectively [17]. Estimated marginal means for the estimated renal function were obtained using multivariate general linear models adjusted for age, weight, height, and SCr according to specific ranges for each factor. McNemar’s test was used to assess significant differences in drug indications between the CG and non-CG formulae. A sample size of 2480 in each group provided 90% power to detect a noninferiority margin difference between the group proportions of 0.01 with a 2.5% significance level for thromboembolism based on the data from phase III trials [1,2,3,4]. Relative hazard ratios for thromboembolism, major bleeding, and combined events in the on-label groups were determined with Cox regression models using the following covariates: age, sex, body weight, comorbidities (congestive heart failure, hypertension, and diabetes mellitus), history of prior thromboembolism, concomitant use of an antiplatelet drug, and SCr level. Firth’s penalized maximum likelihood bias reduction method for the Cox regression was used to obtain hazard ratios in the survival analyses [18]. Statistical analyses were performed using SPSS version 20 (SPSS Institute, Inc., Chicago, IL, USA), SAS version 9.4 (SAS Institute, Ins., Cary, NC, USA), and MedCalc version 15.8 (MedCalc Software, Mariakerke, Belgium). All tests of significance were two tailed, and statistical significance was set at *P* < 0.05. In multiple comparisons between subgroups, the *p*-value was adjusted with the Bonferroni method. 

## 3. Results

### 3.1. Baseline Characteristics

The baseline characteristics of the patients are summarized with respect to anticoagulation drugs and dose in Table 1. Compared with patients who were prescribed warfarin, the patients who received on-label reduced doses of DOAC were older and more likely to be female, with lower body weights and higher rates of comorbidities. Compared with patients who were prescribed warfarin, the patients who received on-label standard doses of DOAC were younger and less likely to be female, with higher body weights, lower rates of comorbidities, less use of antiplatelet drugs, lower SCr levels, smaller left atriums, and higher left ventricular ejection fractions. Dabigatran, rivaroxaban, apixaban, and edoxaban were prescribed for 7.6 ± 7.5, 7.7 ± 6.6, 7.3 ± 5.6, and 9.1 ± 6.4 months, respectively.

### 3.2. Agreement between Different eGFR Calculation Methods

**Figure 2:**
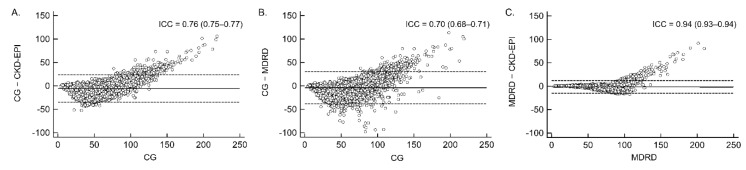
Agreement between formulae in estimating the glomerular filtration rate. Bland–Altman plots represent the mean difference (solid line) and 95% limits of agreement (dashed lines): (**A**) CG and CKD-EPI formulae, (**B**) CG and MDRD formulae, and (C) CKD-EPI and MDRD formulae.

The CG formula exhibited excellent concordance with the CKD-EPI formula (ICC = 0.76) and good concordance with the MDRD formula (ICC = 0.70) in the eGFR results (Figure 2). The overall bias of the CG formula, estimated as the mean difference and standard deviation of differences, was −5.60 ± 14.88 compared with the CKD-EPI formula and −3.99 ± 17.56 compared with the MDRD formula. As the estimated renal function increased, the bias of the CG formula increased positively. The variability in the difference between the CG and MDRD formulae increased as the mean increased. At approximately 50 mL/min, the CG formula was nearly within the 95% limit of agreement (dashed lines in Figure 2). The MDRD and CKD-EPI formulae had excellent concordance (ICC = 0.94) with the lowest bias (−1.60 ± 6.86). 

**Figure 3:**
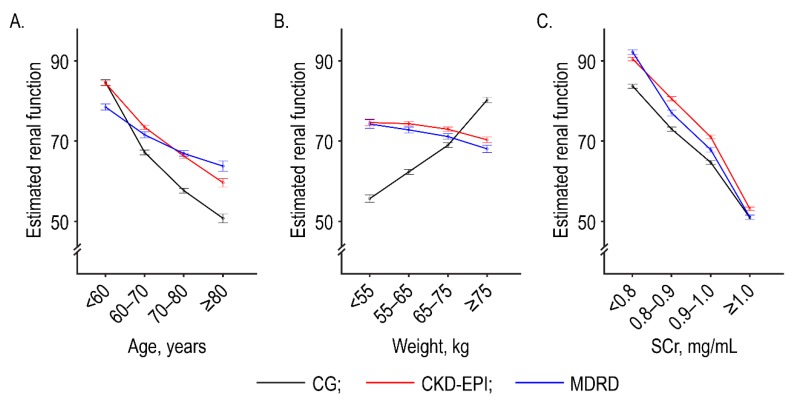
Adjusted marginal means of estimated renal function according to specific age ranges (**A**), weights (**B**), and serum creatinine levels (**C**). Estimated renal function is defined as creatinine clearance (mL/min) in the CG formula and as the estimated glomerular filtration rate (mL/min/1.73 m^2^) in the CKD-EPI and MDRD formulae. Error bars indicate 95% confidence intervals.

After adjusting for potential confounders, we found significant differences in the estimates between the CG and non-CG formulae for specific ranges of age, weight, and SCr (Figure 3). The CG formula underestimated the renal function of underweight and older patients, and it overestimated the renal function of overweight patients, compared with the other formulae. 

### 3.3. Comparison of Drug Indications 

**Figure 4:**
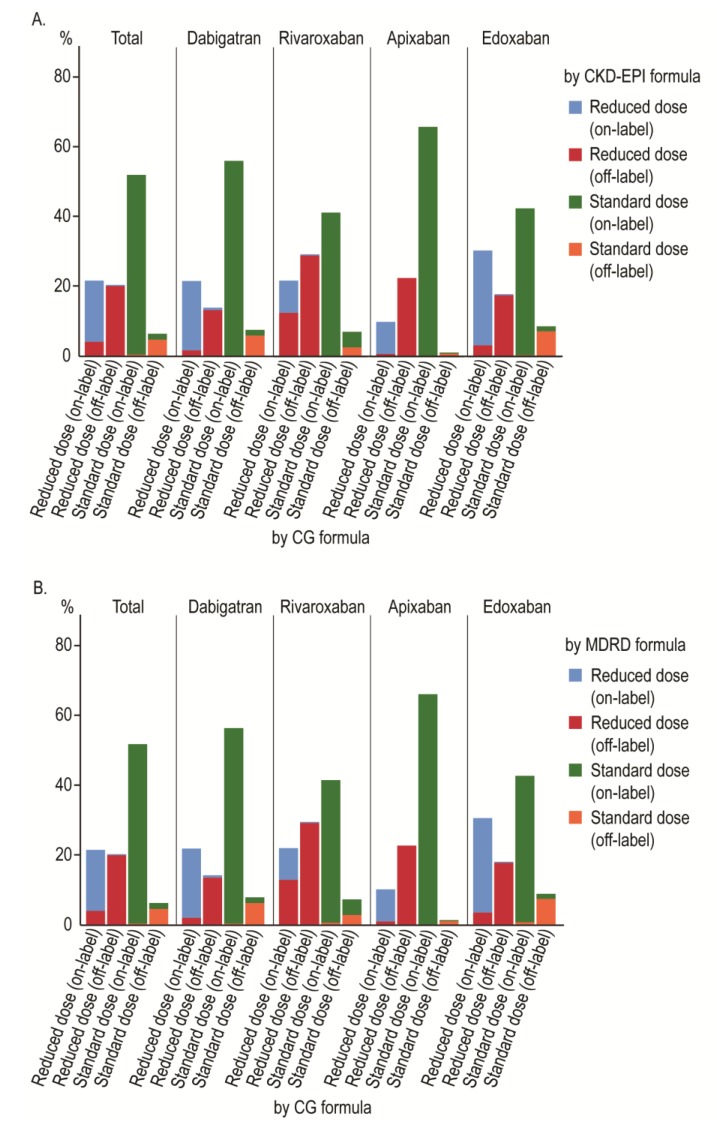
Clustered stacked bar graph showing the concordance of drug indications according to the (**A**) CKD-EPI and (**B**) MDRD formulae.

Figure 4 and Table A2 show the proportions of each drug indication categorized using the different formulae with statistical significances (McNemar’s test). The discordance rate of drug indications between the CG and CKD-EPI formulae was 6.3%. Among different DOACs, rivaroxaban showed the highest discordance rate (17.8%), followed by edoxaban (5.6%), dabigatran (4.5%), and apixaban (1.1%). Among the on-label indications under the CG formula, the discordance rates for the reduced and standard doses were 18.3% and 0.5%, respectively, with the CKD-EPI formula, whereas patients with and without renal impairment with the CG formula (50 mL/min) were recategorized 60.5% and 1.0% of the time, respectively, with the CKD-EPI formula. The results with the MDRD formula were similar to those with the CKD-EPI formula.

### 3.4. Clinical Effectiveness and Safety of On-Label Use According to Different Formulae

**Figure 5:**
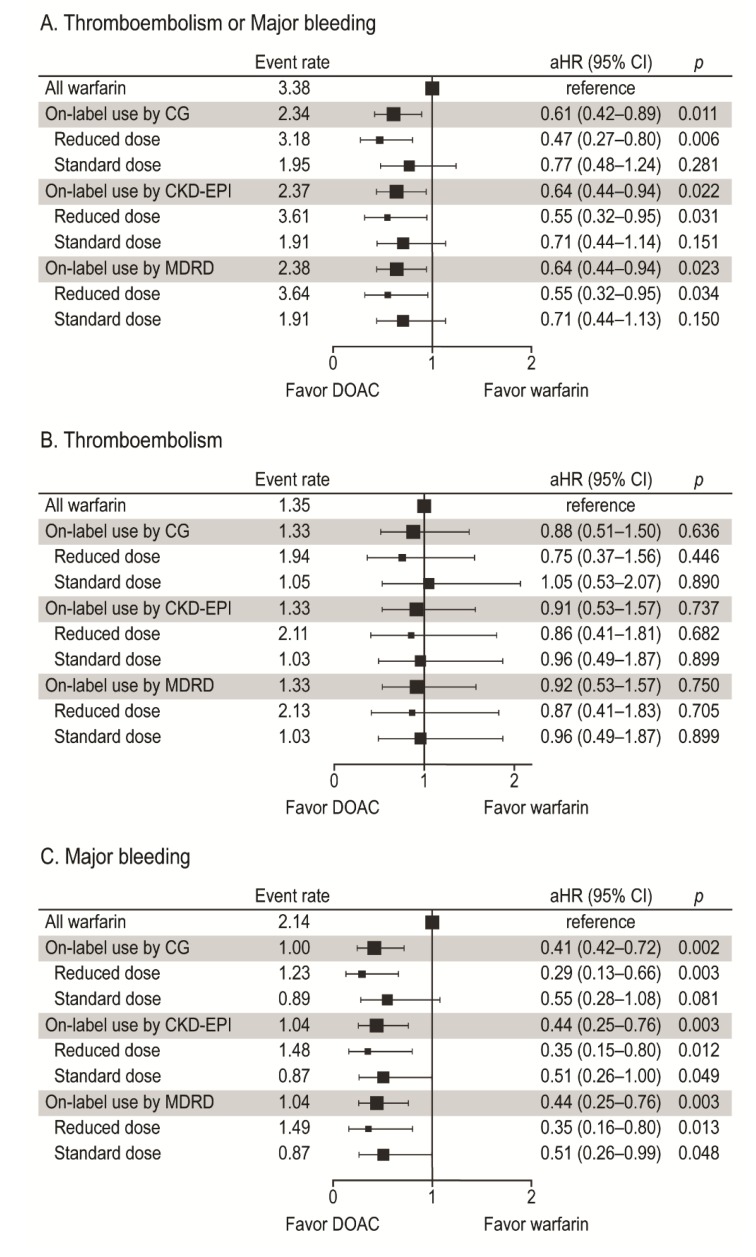
Forest plot of the adjusted hazard ratio and event rate (%/year) for thromboembolism or major bleeding (**A**), thromboembolism (**B**), and major bleeding (**C**). *P* < 0.025 (with the Bonferroni correction) was for subgroup analyses by dose.

During the mean anticoagulation duration of 11.5 ± 11.4 months, a thromboembolism occurred in 24 patients (1.33%/year) in the DOAC group (on-label by the CG formula) versus 47 patients (1.35%/year) in the warfarin group (*P* < 0.001 for noninferiority). In the multivariate Cox proportional hazards regression models, on-label indications, regardless of the formula used, were not associated with a risk of thromboembolism (Figure 5B). However, they were associated with decreased risks of composite and major bleeding compared to warfarin (Figure 5A,C). In the subgroup analysis by dose, a reduced dose was significantly associated with a decreased risk of major bleeding regardless of the formula used (all *Ps* < 0.025 with the Bonferroni correction) (Figure 5C). 

In patients receiving a reduced dose categorized as on-label by the CG formula, a concordant drug indication, defined as an on-label use by a non-CG formula, was associated with a decreased risk of major bleeding compared to warfarin (all *Ps* < 0.017 with the Bonferroni correction), whereas a discordant drug indication carried no significant difference in the risk of adverse events compared to warfarin or a concordant drug indication (Table 2).

## 4. Discussion

### 4.1. Main Findings

To evaluate the clinical utility of the CG formula, we compared the estimated renal function, proportion of drug indications, and clinical outcomes from three formulae. Our main findings are as follows: (1) compared with the CKD-EPI and MDRD formulae, the CG formula generally underestimated the renal function of underweight patients by 4–6 mL/min; (2) an on-label indication for a reduced dose under the CG formula was recategorized as an off-label indication under the non-CG formulae in 18–19% of cases; (3) the risks of a negative clinical outcome were similar among the three formulae, which all showed a lower risk for major bleeding and similar risk for thromboembolism compared to warfarin; (4) discordance in the drug indication with the different formulae was not associated with any risk of an adverse event from a reduced dose. 

### 4.2. Dose Criteria in DOAC Labels 

Because each DOAC’s pharmacokinetic and pharmacodynamic properties are different, their labels include different criteria for age, weight, concomitant drugs, and renal function [19,20,21,22,23]. The volume of distribution, defined as the distribution of a drug between plasma and the rest of the body, determines the effect of weight on drug exposure. Edoxaban is known to have a high volume of distribution, and so patients who are underweight might have a higher exposure to edoxaban than to other DOACs [6]. Reliance on the CrCl clause in the criteria could affect the discordance rate of drug indications with the different formulae. In the present study, the discordance rate was the highest for rivaroxaban, which depended on a sole CrCl criterion, and lowest for apixaban. 

### 4.3. Performance of Formulae in Estimating Renal Function

Because direct measurement of GFR using exogenous filtration markers is cumbersome in clinical practice, indirect estimation of GFR using renal formulae and an endogenous filtration marker, such as a creatinine, is recommended when assessing renal function. However, the accuracy of estimated renal function is affected by the formulae used and each patient’s condition. A formula using SCr has limited value in the early stage of renal injury and at a high GFR level because the SCr level rises after a significant loss of renal function and is changed by factors other than GFR, such as creatinine production and extrarenal excretion. Recently, the assay used to determine SCr has been standardized to minimize the variation across laboratories. We found that the CrCl from the CG formula was lower than the eGFRs from the CKD-EPI and MDRD formulae in older and underweight patients, similar to previous studies [24,25]. The CKD-EPI and MDRD formulae were more accurate than the CG formula across the GFR range and in diverse populations [11,26,27]. Although suggested modifications with ethnic coefficients improved the performance of the CKD-EPI and MDRD formulae in Chinese, Japanese, and Korean people, those modified formulae have not yet been validated in other countries [28,29,30]. 

### 4.4. Clinical Use of the Cockcroft-Gault Formula for Dosing DOAC

Several issues affect the accuracy of the CG formula’s estimation of renal function. First, the CG formula was developed using non-standardized SCr [31]. Previously, non-standardized SCr had significant interlaboratory variability because of the many different assays used to measure it. After the standardized assay for SCr was instituted, the MDRD formula was modified to use it, and the CKD-EPI formula was developed [9,11]. Second, the CG formula is not corrected for body surface area (BSA). Because BSA is closely related to the metabolic rate, correction for BSA could increase the accuracy of the estimated value and allow comparison of those data across body sizes [32,33,34]. However, poor correlation between BSA and measured GFR has been reported [35]. Correction for BSA also failed to eliminate the dependency of GFR on BSA [35,36] and resulted in the underestimation of renal function in obese patients [37]. Third, the accuracy of the CG formula is significantly affected by age and weight, overestimating renal function in obese patients and underestimating it in elderly and underweight patients [24]. However, patients receiving the standard dose were rarely recategorized as receiving an off-label standard dose by the non-CG formulae because the difference in the estimates was small. Furthermore, the plasma concentration of a DOAC is not determined by renal clearance alone. Other pharmacokinetic variables, such as drug interactions, hepatic function, and body weight, are also important. Although it is acknowledged that the CG formula underestimates renal function in patients with low body weight, low body weight is also associated with higher drug exposure to all the DOACs except rivaroxaban [38,39,40,41]. Thus, the CG formula could reliably reflect the pharmacokinetic behavior of DOACs in patients with low body weight and intermediate renal function.

### 4.5. Limitations

First, this study was conducted at a single tertiary referral center in a single country with few ethnicities. The value of extrapolating these results to other regions with different labels and ethnicities might be limited. Second, no observational study can establish the causality of clinical events because of residual confounding and reverse causation. Third, the total number of patients might be too small to adequately analyze the subgroups with discordant DOAC indications because adverse events occurred rarely. Fourth, we included not only patients who were prescribed an anticoagulant as the first drug but also those who switched to another anticoagulant, which might have increased the rate of adverse events (because patients who experience an adverse event on one drug often change to another drug) compared with the phase III trials, which considered only anticoagulation-naive patients. To minimize selection bias, we applied our inclusion criteria equally to the warfarin and DOAC groups. Fifth, the results from the individual DOAC groups composed of patients taking one of the four DOACs might have depended on their respective proportions because the indications for each specific drug were different.

## 5. Conclusions

The CG formula does have an increasing bias in estimating renal function as patients become older, are underweight, or have nearly normal renal function, which can increase the discordance rate in reduced dose patients (but not standard dose patients). However, the differences in estimated renal function level and categorized dose indications are unlikely to affect the risk of thromboembolism or major bleeding in on-label use of a DOAC. Thus, the application of an algorithm the risk–benefit profile of which is known is more important than the choice of renal formula.

## Figures and Tables

**Table 1:** Baseline characteristics.

	Warfarin (N = 2659)	Direct Oral Anticoagulant					
		On-Label by CG		On-Label by CKD-EPI		On-Label by MDRD	
		Reduced Dose (*n* = 777)	Standard Dose (*n* = 1873)	Reduced Dose (*n* = 647)	Standard Dose (*n* = 1925)	Reduced Dose (*n* = 645)	Standard Dose (*n* = 1923)
Age, years	65.3 ± 11.9	77.3 ± 8.1 *	62.4 ± 10.7 *	77.0 ± 8.5 *	62.8 ± 10.9 *	77.0 ± 8.5 *	62.8 ± 10.9 *
Female	820 (30.8)	474 (61.0) *	496 (26.5) *	395 (61.1) *	522 (27.1) *	393 (60.9) *	521 (27.1) *
Weight, kg	67.7 ± 12.1	56.8 ± 9.7 *	71.0 ± 11.2 *	57.1 ± 10.5 *	70.5 ± 11.3 *	57.1 ± 10.5 *	70.5 ± 11.2 *
Hypertension	1980 (74.5)	650 (83.7) *	1315 (70.2) *	537 (83.0) *	1354 (70.3) *	535 (82.9) *	1352 (70.3) *
Diabetes	717 (27.0)	239 (30.8) *	452 (24.1) *	212 (32.8) *	463 (24.1) *	210 (32.6) *	462 (24.0) *
Congestive heart failure	865 (32.5)	303 (39.0) *	282 (15.1) *	249 (38.5) *	303 (15.7) *	248 (38.4) *	302 (15.7) *
Any prior thromboembolism	600 (22.6)	311 (40.0) *	414 (22.1)	253 (39.1) *	443 (23.0)	252 (39.1) *	443 (23.0)
CHA_2_DS_2_-VASc score **	2.8 ± 1.9	4.5 ± 1.6 *	2.3 ± 1.7 *	4.4 ± 1.6 *	2.4 ± 1.7 *	4.4 ± 1.6 *	2.4 ± 1.7 *
Concomitant antiplatelet drug	296 (11.1)	70 (9.0)	65 (3.5) *	61 (9.4)	70 (3.6) *	60 (9.3)	71 (3.7) *
Serum creatinine, mg/mL	1.11 ± 0.75	1.09 ± 0.43	0.96 ± 0.19 *	1.11 ± 0.45	0.96 ± 0.19 *	1.11 ± 0.45	0.96 ± 0.19 *
Left ventricular ejection fraction, %	51.6 ± 10.0	51.9 ± 10.0	53.2 ± 7.9 *	52.1 ± 9.9	53.1 ± 8.0 *	52.0 ± 9.9	53.1 ± 8.0 *
Left atrial diameter, mm	44.6 ± 7.1	44.7 ± 7.7	43.2 ± 6.4 *	44.9 ± 8.0	43.2 ± 6.5 *	44.8 ± 8.0	43.2 ± 6.5 *
Drug adherence, %	92.0 ± 17.0	91.9 ± 17.9	90.8 ± 19.9 *	91.4 ± 18.7	90.7 ± 20.1 *	91.4 ± 18.7	90.7 ± 20.1 *
≥80% (adherent)	2152 (87.6)	595 (88.1)	1384 (85.1) *	492 (87.5)	1429 (85.2) *	490 (87.5)	1426 (85.2) *

Data are presented as the mean ± standard deviation or number (%). * *P* < 0.05 compared to the overall warfarin group. ** One point each for congestive heart failure, hypertension, age of 65–74 years, diabetes mellitus, and vascular disease (myocardial infarction or peripheral arterial disease), and two points for age of 75 years or older and a previous stroke.

**Table 2:** Clinical outcomes with a reduced dose of a direct oral anticoagulant according to the concordance of the drug indication between the Cockcroft-Gault formula and other formulae.

	Event Rate (%/year)		Adjusted HR (95% CI) *	P **
	DOAC	Warfarin		
Thromboembolism				
Concordant (on-label by CG and on-label by CKD-EPI) vs. Warfarin	1.95	1.35	0.75 (0.34–1.63)	0.465
Discordant (on-label by CG and off-label by CKD-EPI) vs. Warfarin	1.90	1.35	0.80 (0.19–3.41)	0.759
Discordant vs. Concordant (CG and CKD-EPI)			1.39 (0.29–6.69)	0.680
Concordant (on-label by CG and on-label by MDRD) vs. Warfarin	1.97	1.35	0.76 (0.35–1.65)	0.482
Discordant (on-label by CG and off-label by MDRD) vs. Warfarin	1.83	1.35	0.75 (0.18–3.23)	0.704
Discordant vs. Concordant (CG and MDRD)			1.21 (0.25–5.82)	0.811
Major bleeding				
Concordant (on-label by CG and on-label by CKD-EPI) vs. Warfarin	1.51	2.14	0.36 (0.16–0.81)	0.014
Discordant (on-label by CG and off-label by CKD-EPI) vs. Warfarin	0.00	2.14	0.59 (0.16–2.18)	0.432^†^
Discordant vs. Concordant (CG and CKD-EPI)			0.82 (0.08–7.94)	0.862^†^
Concordant (on-label by CG and on-label by MDRD) vs. Warfarin	1.53	2.14	0.36 (0.16–0.82)	0.015
Discordant (on-label by CG and off-label by MDRD) vs. Warfarin	0.00	2.14	0.59 (0.17–2.13)	0.423^†^
Discordant vs. Concordant (CG and MDRD)			0.67 (0.07–6.35)	0.730^†^

* Adjusted for age, sex, body weight, comorbidities (congestive heart failure, hypertension, diabetes mellitus, prior thromboembolism), concomitant use of an antiplatelet drug, and serum creatinine. ** *P*-value required for statistical significance was <0.017 (with the Bonferroni correction). † Firth’s bias reduction method used because of no event.

## Appendix A

**Table A1:** Guidelines for dosing direct oral anticoagulants according to renal function.

Drug	Dosage and Administration *
Dabigatran	150 mg twice daily 110 mg twice daily 30≤ serum creatinine clearance (CrCl) <50 mL/min Body weight ≤50 kg Age ≥75 years Concomitant potent P-glycoprotein inhibitor therapy^†^
Rivaroxaban	20 mg once daily 15 mg once daily 15≤ serum CrCl <50 mL/min
Apixaban	5 mg twice daily 2.5 mg twice daily any 2 of age ≥80 years, body weight ≤60 kg, or serum creatinine ≥1.5 mg/dL 15≤ serum CrCl <30 mL/min
Edoxaban	60 mg once daily 30 mg once daily 15≤ serum CrCl <50 mL/min Body weight ≤60 kg Concomitant potent P-glycoprotein inhibitor therapy^‡^

* Determined based on randomized controlled trials except for dabigatran. † included verapamil, cyclosporin, tacrolimus, dronedarone, and itraconazole in this study. ‡ included cyclosporin, tacrolimus, dronedarone, and itraconazole in this study.

**Table A2:** Comparison (via McNemar’s test) of drug indications based on the glomerular filtration rate estimated with different formulae.

	By CG		By CKD-EPI				*p*	By MDRD				*p*
			Reduced		Standard			Reduced		Standard		
			On	Off	On	Off		On	Off	On	Off	
All DOACs	Reduced	On	635	142	0	0	<0.05	633	144	0	0	<0.05
		Off	12	721	0	0		12	721	0	0	
	Standard	On	0	0	1863	10		0	0	1861	12	
		Off	0	0	62	164		0	0	62	164	
Dabigatran	Reduced	On	169	16	0	0	<0.05	169	16	0	0	<0.05
		Off	6	114	0	0		6	114	0	0	
	Standard	On	0	0	476	2		0	0	476	2	
		Off	0	0	14	52		0	0	14	52	
Rivaroxaban	Reduced	On	58	79	0	0	<0.05	57	80	0	0	<0.05
		Off	2	182	0	0		2	182	0	0	
	Standard	On	0	0	257	2		0	0	256	3	
		Off	0	0	28	17		0	0	28	17	
Apixaban	Reduced	On	89	8	0	0	<0.05	89	8	0	0	<0.05
		Off	0	218	0	0		0	218	0	0	
	Standard	On	0	0	636	0		0	0	636	0	
		Off	0	0	3	9		0	0	3	9	
Edoxaban	Reduced	On	319	39	0	0	<0.05	318	40	0	0	<0.05
		Off	4	207	0	0		4	207	0	0	
	Standard	On	0	0	494	6		0	0	493	7	
		Off	0	0	17	86		0	0	17	86	

Abbreviations: CG, Cockcroft-Gault formula; CKD-EPI, Chronic Kidney Disease Epidemiology Collaboration formula; DOAC, direct oral anticoagulant; MDRD, Modification of Diet in Renal Disease Study formula; Off, off-label; On, on-label.
